# Total arch replacement in octogenarians

**DOI:** 10.1093/icvts/ivab256

**Published:** 2021-09-22

**Authors:** Kohei Hachiro, Takeshi Kinoshita, Tomoaki Suzuki, Tohru Asai

**Affiliations:** Division of Cardiovascular Surgery, Department of Surgery, Shiga University of Medical Science, Otsu, Shiga, Japan; Division of Cardiovascular Surgery, Department of Surgery, Shiga University of Medical Science, Otsu, Shiga, Japan; Division of Cardiovascular Surgery, Department of Surgery, Shiga University of Medical Science, Otsu, Shiga, Japan; Division of Cardiovascular Surgery, Department of Surgery, Shiga University of Medical Science, Otsu, Shiga, Japan

**Keywords:** Total arch replacement, Octogenarians

## Abstract

**OBJECTIVES:**

We investigated the effect of a preoperative age ≥80 years on postoperative outcomes in patients who underwent isolated elective total arch replacement using mild hypothermic lower body circulatory arrest with bilateral antegrade selective cerebral perfusion.

**METHODS:**

A total of 140 patients who had undergone isolated elective total arch replacement between January 2007 and December 2020 were enrolled in the present study. We compared postoperative outcomes between 30 octogenarian patients (≥80 years old; Octogenarian group) and 110 non-octogenarian patients (≤79 years old; Non-Octogenarian group).

**RESULTS:**

Overall 30-day mortality and hospital mortality were 0% in both groups, and there was no significant difference in overall survival between the 2 groups (log-rank test, *P* = 0.108). Univariable Cox proportional hazard analysis showed that age as continuous variable was only the predictor of mid-term all-cause death (hazard ratio 1.08, 95% confidence interval 1.01–1.16; *P* = 0.037), but not in the Octogenarians subgroup (*P* = 0.119).

**CONCLUSIONS:**

Preoperative age ≥80 years is not associated with worse outcomes postoperatively after isolated elective total arch replacement with mild hypothermic lower body circulatory arrest and bilateral antegrade selective cerebral perfusion.

**Figure:**
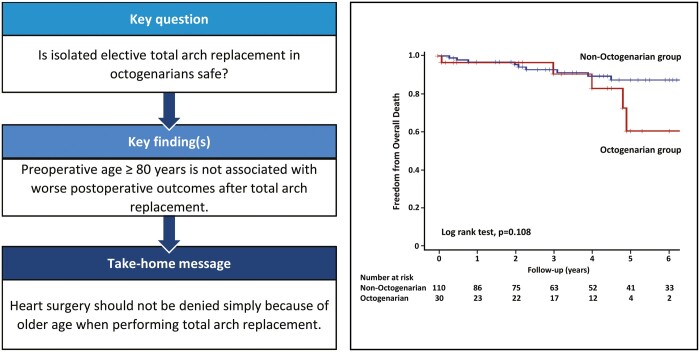

## INTRODUCTION

Progress in perioperative management and surgical techniques, including antegrade selective cerebral perfusion (SCP), has improved outcomes after total arch replacement (TAR) [1–3]. However, TAR remains a challenging operation because of coagulopathy and multiorgan disorders associated with hypothermic circulatory arrest and prolonged use of cardiopulmonary bypass (CPB).

The number of older patients who undergo major surgery is increasing. These patients often present with multiple comorbidities and unique physiology in the respiratory, cardiovascular and metabolic systems [4, 5]. A previous study reported that the mortality rate was 13.0% in octogenarians undergoing thoracic aortic surgery [6]. However, even with expanded indications for endovascular treatment for aortic arch aneurysm, conventional TAR remains an important option. We retrospectively investigated the postoperative outcomes in octogenarians undergoing isolated elective TAR.

## PATIENTS AND METHODS

The study was approved by our institutional review board in Shiga University of Medical Science (approval no. R2019-279, 8 January 2020) and written patients informed consent was waived by the institutional review board.

**Figure 1:**
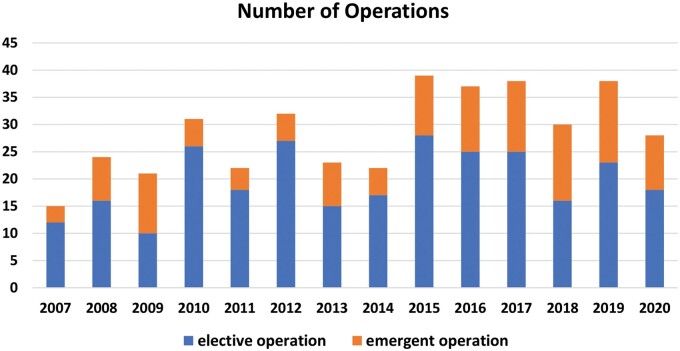
Number of total arch replacement operations in our institution.

**Figure 2:**
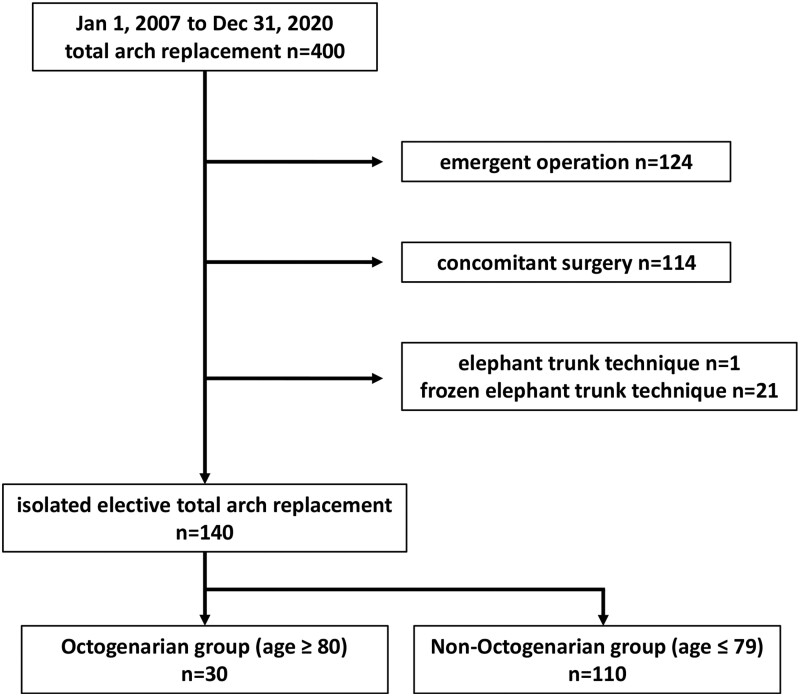
Patient population in this study.

Between January 2007 and December 2020, 400 consecutive patients underwent TAR at the Shiga University of Medical Science and 276 patients were performed elective operation (Fig. 1). We excluded 114 patients who underwent concomitant surgery including valve surgery, coronary artery bypass grafting, anti-arrhythmic surgery and so on. In addition, after 1 patient who underwent elephant trunk technique and 21 patients who underwent frozen elephant trunk technique were excluded, 140 patients were enrolled in the present study (Fig. 2). We compared clinical results between 30 octogenarian patients (≥80 years old; Octogenarian group) and 110 non-octogenarian patients (≤79 years old; Non-Octogenarian group).

We performed ultrasonography of carotid arteries and magnetic resonance angiography of the carotid, vertebral and intracranial arteries to evaluate potential cerebral ischaemia. Preoperative coronary computed tomography angiography or invasive coronary angiography were performed to evaluate coronary artery disease. We measured the distance between the skin and distal anastomosis from the postoperative computed tomography image and did not routinely evaluate frailty and cognition function before surgery.

### Outcome measures and definitions

The primary outcome measure was all-cause mortality. Cardiac death included death caused by myocardial infarction, heart failure or lethal arrhythmia. Documentation of the cause of death was based on information obtained from witnesses, family members, death certificates, hospital records and autopsy records. The secondary measure was in-hospital outcome, including mortality, stroke and paraplegia. Stroke was defined as newly developed paralysis symptoms of the central nervous system remaining for ≥24 h. Transient ischaemic attack was defined as a sudden, focal neurological deficit of presumed vascular origin lasting <24 h. Paraplegia was defined as newly developed numbness, weakness or bilateral sensory dysfunction of the lower extremities. All patients’ data were collected prospectively and entered in our database. The patients after TAR undergo computed tomography once a year in our hospital. Data for all preoperative, intraoperative and postoperative variables were obtained from the database or directly from the electronic medical records at our hospital.

### Surgical treatment

In our institution, we perform thoracic aortic surgery on patients with fusiform aneurysms of 55 mm or more, saccular aneurysms and aneurysms with an expansion rate of 5 mm or more in half a year. We perform TAR for aortic arch aneurysm regardless of age without performing debranching thoracic endovascular aortic repair. In case of distal arch aneurysm, we have started to use frozen elephant trunk procedure since 2015. However, due to concerns about stent graft induced new entry or spinal cord ischaemia, we continue to perform conventional TAR if possible.

Patients were placed in the supine position and anaesthesia was maintained in the standard manner. Transoesophageal echocardiography was used to confirm cardiac and valvular function, and temperature probes were placed for tympanic membrane, rectal and bladder body temperatures. The details of TAR performed at our institution have been reported previously [2, 7]. Briefly, the approach for TAR in all patients was through median sternotomy. The arterial cannulation site was determined using preoperative computed tomography and intraoperative epiaortic ultrasonographic findings. Myocardial protection was performed by retrograde intermittent infusion of a cold blood cardioplegic solution that was introduced directly to the coronary sinus via right atriotomy. Systemic cooling was considered adequate for circulatory arrest when the tympanic temperature fell to 25°C and the core temperature fell to <30°C, followed by SCP with balloon-tipped catheters. A 14-Fr balloon-tipped cannula was inserted into brachiocephalic artery and 12-Fr cannula into the left common carotid, and left subclavian arteries. Three brachiocephalic branches are divided at the level of healthy arterial wall. If any atheromatous emboli are expected, a short period of retrograde cerebral perfusion is performed prior to SCP. When left vertebral artery arose from the arch, 6- or 10-Fr SCP cannula was inserted. SCP flow was 10–12 ml/kg/min with a perfusate temperature of 25–28°C. The brain oxygen saturation is monitored using INVOS 5100C (Somanetics, Troy, MI, USA). According to the judgement of the surgeon, J-Graft (Japan Lifeline, Tokyo, Japan), Triplex (Terumo, Tokyo, Japan) or Gelweave (Vascutek Ltd, Scotland, UK) were used for the reconstruction of aortic arch vessels. In all cases, we used four-branched graft. Anastomoses were sequentially constructed at the distal arch, proximal root, left subclavian artery, left carotid artery and right brachiocephalic artery. All these vessels are anastomosed to vascular prosthesis in an end-to-end fashion with monofilament continuous suture without using island technique. When left vertebral artery arose from the arch, that was anastomosed to the vascular prosthesis which was anastomosed to left subclavian artery in an end-to-side fashion. An ESTECH retractor was often used during the distal procedure to create a comfortable surgical field especially in case of large distal arch aneurysm. After completing distal anastomosis, a vascular prosthesis was clamped proximally, antegrade systemic circulation was restarted through the side branch of the prosthesis, and rewarming of the whole body began.

### Statistical analysis

Continuous variables are presented as mean ± standard deviation or median and interquartile range, while categorical variables are presented as number and percentage. Comparisons of patients’ clinical characteristics between the 2 groups were performed using the unpaired *t*-test for normally distributed variables, the Mann–Whitney *U*-test for skewed variables and Pearson’s *χ*^2^ test for categorical variables.

The Kaplan–Meier method was used to describe survival rates and the log-rank test was used for comparisons. Univariable and multivariable logistic regression analyses were performed to identify the independent predictors of postoperative stroke. We also performed univariable and multivariable Cox proportional hazard regression analyses to analyse all-cause death. Variables reaching *P*-value <0.050 in univariable analysis or those that were considered clinically important were entered into multivariable analysis. All statistical testing was two-sided. Absolute standardized mean differences were calculated to compare the balance in base line characteristics between the Octogenarian and Non-Octogenarian groups. Results were considered statistically significant at *P*-value <0.050, and all statistical analyses were performed using the IBM Statistical Package for Social Sciences, version 25.0 (IBM Corp., Armonk, NY, USA).

## RESULTS

The preoperative characteristics of each group are shown in Table 1. The mean age of our study population was 72.3 ± 10.0 years and consisted of 116 (82.9%) men. Body mass index was significantly higher in the Non-Octogenarian group than in the Octogenarian group (*P* = 0.006), and there was no patient whose body mass index was >30 kg/m^2^ in either groups. Non-Octogenarian group had significantly more peripheral artery disease (*P* < 0.001) and history of cardiovascular surgery. No patients had a left ventricular ejection fraction <40% in either group. Octogenarian group had larger aneurysms (*P* = 0.002) and more atherosclerotic aneurysms (*P* = 0.031).

**Table 1:** Preoperative patient characteristics

	Octogenarian (*n* = 30)	Non-octogenarian (*n* = 110)	*P*-value	ASMD
Age (years), mean ± SD	83.2 ± 2.3	69.3 ± 9.1	<0.001	2.094
Sex (male), *n* (%)	24 (80.0)	92 (83.6)	0.642	0.093
Body mass index (kg/m^2^), mean ± SD	21.7 ± 2.7	23.3 ± 2.8	0.006	0.582
Hypertension, *n* (%)	24 (80.0)	85 (77.3)	0.752	0.066
Diabetes mellitus, *n* (%)	9 (30.0)	25 (22.7)	0.414	0.166
Dyslipidaemia, *n* (%)	13 (43.3)	39 (35.5)	0.432	0.160
Smoking history, *n* (%)	19 (63.3)	80 (72.7)	0.320	0.203
COPD, *n* (%)	3 (10.0)	10 (9.1)	0.880	0.031
Previous CVD, *n* (%)	7 (23.3)	18 (16.4)	0.381	0.174
Peripheral artery disease, *n* (%)	0 (0)	15 (13.6)	< 0.001	0.561
Atrial fibrillation, *n* (%)	1 (3.3)	1 (0.9)	0.325	0.168
Insuline therapy, *n* (%)	0 (0)	2 (1.8)	0.461	0.191
LVEF <40%, *n* (%)	0 (0)	0 (0)		
eGFR (ml/min/1.73 m^2^), mean ± SD	55.2 ± 20.9	62.5 ± 26.9	0.171	0.303
eGFR <60 ml/min/1.73 m^2^, *n* (%)	16 (53.3)	57 (51.8)	0.884	0.030
Haemodialysis, *n* (%)	2 (6.7)	0 (0)	0.161	0.379
Connective tissue disease, *n* (%)	0 (0)	2 (1.8)	0.461	0.191
Previous sternotomy, *n* (%)	1 (3.3)	18 (16.4)	0.009	0.451
Previous thoracic aortic surgery, *n* (%)	0 (0)	12 (10.9)	< 0.001	0.495
Previous CABG, *n* (%)	0 (0)	4 (3.6)	0.045	0.273
Aneurysm diameter (mm), mean ± SD	62.4 ± 9.5	55.6 ± 7.4	0.002	0.799
Aneurysm type, *n* (%)				
Atherosclerotic aneurysm	23 (76.7)	62 (56.4)	0.031	0.441
Penetrating aortic ulcer	4 (13.3)	23 (20.9)	0.312	0.203
Chronic dissection	3 (10.0)	25 (22.7)	0.069	0.349
Aortitis	0 (0)	0 (0)		
EuroSCORE II (%), median (IQR)	3.1 (2.4–4.3)	2.2 (1.8–3.5)	0.192	0.300

ASMD: absolute standardized mean difference; CABG: coronary artery bypass grafting; COPD: chronic obstructive pulmonary disease; CVD: cerebrovascular disease; eGFR: estimated glomerular filtration rate; EuroSCORE: European System for Cardiac Operative Risk Evaluation; IQR: interquartile range; LVEF: left ventricular ejection fraction; SD: standard deviation.

### Early outcomes

**Figure 3:**
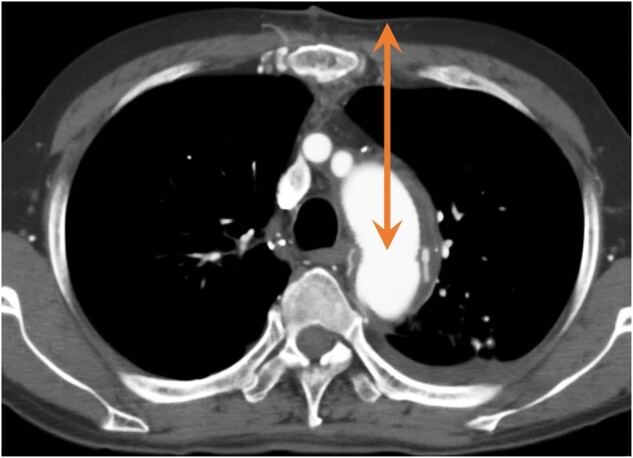
Distance between the skin and distal anastomosis.

Table 2 shows the operative and postoperative outcomes. The Octogenarian group had a significantly lower circulatory arrest time, higher minimum core temperature and a shorter distance between the skin and distal anastomosis than the Non-Octogenarian group (Fig. 3). Three patients (10.0%) in the Octogenarian group and 6 patients (5.5%) in the Non-Octogenarian group had the left vertebral artery that arose from the arch. No patients developed postoperative myocardial infarction or paraplegia in either group. There were no significant differences in postoperative complications, including stroke and prolonged ventilation. Both of the 2 patients who underwent postoperative stroke discharged with modified Rankin Scale score of 2. Overall 30-day mortality and hospital mortality were 0% in both groups. We included preoperative data, operative data, age ≥80 (years) and age (years) into logistic regression analysis of postoperative stroke. However, independent predictors of postoperative stroke could not be calculated because of the small number of patients (*n* = 2).

**Table 2:** Operative and postoperative data

	Octogenarian (*n* = 30)	Non-octogenarian (*n* = 110)	*P*-value	ASMD
Operative data				
Operation time (m), mean ± SD	220 ± 61	244 ± 65	0.073	0.381
CPB time (m), mean ± SD	124 ± 34	138 ± 38	0.069	0.388
SCP time (m), mean ± SD	88 ± 26	93 ± 25	0.285	0.221
Circulatory arrest time (m), mean ± SD	45 ± 13	52 ± 23	0.044	0.375
Minimum core temperature (°C), mean ± SD	25.9 ± 2.1	24.8 ± 2.2	0.027	0.511
Distance between skin and distal anastomosis (cm), mean ± SD	8.9 ± 1.7	10.5 ± 1.7	<0.001	0.941
Cannulation, *n* (%)				
Ascending	29 (96.7)	98 (89.1)	0.208	0.299
Femoral	0 (0)	3 (2.7)	0.364	0.236
Axillary	1 (3.3)	9 (8.2)	0.364	0.212
Postoperative data				
Myocardial infarction, *n* (%)	0 (0)	0 (0)		
Stroke, *n* (%)	1 (3.3)	1 (0.9)	0.325	0.168
Transient ischaemic attack, *n* (%)	0 (0)	0 (0)		
Paraplegia, *n* (%)	0 (0)	0 (0)		
Pneumonia, *n* (%)	2 (6.7)	1 (0.9)	0.232	0.307
Atrial fibrillation, *n* (%)	5 (16.7)	15 (13.6)	0.677	0.087
Sepsis, *n* (%)	0 (0)	1 (0.9)	0.603	0.135
Re-exploration for bleeding, *n* (%)	1 (3.3)	3 (2.7)	0.861	0.035
Ventilation >48 h, *n* (%)	1 (3.3)	1 (0.9)	0.325	0.168
ICU stay > 48 h, *n* (%)	4 (13.3)	5 (4.5)	0.193	0.313
Gastrointestinal bleeding, *n* (%)	0 (0)	1 (0.9)	0.603	0.135
Continuous haemodiafiltration, *n* (%)	2 (6.7)	1 (0.9)	0.232	0.307
Recurrent nerve paralysis, *n* (%)	5 (16.7)	23 (21.1)	0.595	0.113
30-Day mortality, *n* (%)	0 (0)	0 (0)		
Hospital mortality, *n* (%)	0 (0)	0 (0)		
Length of hospital stay (days), mean ± SD	21.5 ± 9.4	24.6 ± 15.5	0.309	0.242

ASMD: absolute standardized mean difference; CPB: cardiopulmonary bypass; ICU: intensive care unit; SCP: selective cerebral perfusion; SD: standard deviation.

### Mid-term outcomes

**Figure 4:**
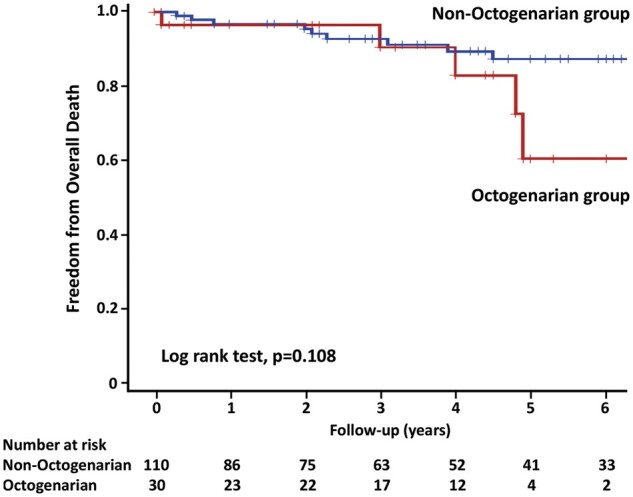
Freedom from overall death.

Follow-up was completed in 94.3% (132/140) of the patients and the mean follow-up duration was 3.8 ± 3.1 years (maximum: 13.3 years). All causes of death are shown in Table 3. There were 5 deaths in the Octogenarian group and 10 in the Non-Octogenarian group. No patients died because of cardiac problems in either group. There was no significant difference in non-cardiac death between the 2 groups, including pneumonia, stroke and renal failure. The adjusted 4-year estimated rate of freedom from overall death was 83% in the Octogenarian group and 89.5% in the Non-Octogenarian group (Fig. 4), with no significant difference in the survival curve between the groups (*P* = 0.108). We analysed univariable Cox proportional hazard analysis for all-cause mortality after preoperative atrial fibrillation (*n* = 2), insulin therapy (*n* = 2), haemodialysis, connective tissue disease (*n* = 2) and history of coronary artery bypass grafting (*n* = 4) were excluded from predictive factors because of the small number of patients. Univariable analysis revealed that age as continuous variable was only the predictor [hazard ratio (HR) 1.08, 95% confidence interval (CI) 1.01–1.16; *P* = 0.037] and age ≥80 was not the predictor (HR 2.39, 95% CI 0.80–7.15; *P* = 0.119), so we could not perform multivariable analysis.

**Table 3:** Causes of overall death

	Octogenarian (*n* = 30), *n* (%)	Non-octogenarian (*n* = 110), *n* (%)	*P*-value	ASMD
All-cause death	5 (16.7)	10 (9.1)	0.315	0.228
Cardiac death	0 (0)	0 (0)		
Myocardial infarction	0 (0)	0 (0)		
Heart failure	0 (0)	0 (0)		
Arrhythmia	0 (0)	0 (0)		
Non-cardiac death	5 (16.7)	10 (9.1)	0.315	0.228
Pneumonia	1 (3.3)	2 (1.8)	0.614	0.095
Stroke	0 (0)	1 (0.9)	0.603	0.135
Sepsis	2 (6.7)	2 (1.8)	0.320	0.245
Cancer	0 (0)	2 (1.8)	0.461	0.191
Renal failure	0 (0)	1 (0.9)	0.603	0.135
Trauma	0 (0)	1 (0.9)	0.603	0.135
Unknown	2 (6.7)	1 (0.9)	0.232	0.307

ASMD: absolute standardized mean difference.

## DISCUSSION

Hybrid arch repair and debranching thoracic endovascular aortic repair are important treatment options in the management of aortic arch aneurysm, especially in elderly patients. There procedures are minimally invasive, but the incidence of postoperative stroke remains high with 5.6–10.6% [8–10] compared to that in open arch repair with 2.6–3.5% [10–12]. In octogenarians with a limited life expectancy, it is important to minimize the postoperative early complication such as stroke that reduces quality of life. In our entire cohort, early mortality was 0% (0/140) and postoperative stroke rate was 1.4% (2/140), which was acceptable outcome. Even in elderly patients, conventional TAR as a one-stage radical treatment for aortic arch aneurysm may need to be reviewed in the era of endovascular aortic repair.

One of the major findings of the present study is that there was no significant difference in 30-day mortality or hospital mortality between the 2 groups. Postoperative early outcomes in the Octogenarian group, which should be at high risk, were not poor. There are 2 possible explanations about this outcome. One is the difference in patient characteristics between both groups. In the Octogenarians group, there were significantly fewer patients who had peripheral artery disease and experienced previous cardiac surgery (Table 1). This may have had a protective effect on the postoperative outcomes in the Octogenarians group. The other is the fact that the time associated with surgery was shorter in the Octogenarian group than in the Non-Octogenarian group. The Octogenarian group tended to have a shorter operation time and CPB time and had a significantly shorter circulatory arrest time (Table 2). Previous reports showed that the CPB time [13, 14] and circulatory arrest time [15] were associated with hospital mortality in aortic arch surgery. Therefore, the shortness of time associated with surgery may have resulted in the good postoperative outcomes in the Octogenarian group. There are some possible reasons that affected the procedure duration.

The Non-Octogenarian group had significantly more patients who underwent previous cardiovascular surgery (Table 1). Off course, adhesions associated with previous surgery makes the operation difficult, which seems to have affect the outcome. We also consider circulatory arrest time to be an important factor influencing surgery time. The core temperature continues to slowly fall after circulatory arrest and it begins to rise as the systemic circulation is restarted [16]. Therefore, the minimum core temperature decreases as the circulatory arrest time increases. In the present study, the Non-Octogenarian group had a significantly longer circulatory arrest time than the Octogenarian group, which may have resulted in a lower minimum core temperature in the Non-Octogenarian group. If the minimum core temperature is low, it takes time to rewarm, which leads to an increase in CPB and operation times. In our institutional method, the circulatory arrest time represents the time of trimming the distal anastomotic site and performing distal anastomosis. Postoperative computed tomography showed that the distance between the skin and distal anastomosis was significantly longer in the Non-Octogenarian group than in the Octogenarian group. Trimming the distal anastomotic site and performing distal anastomosis become more technically difficult as the distance increases. Therefore, the finding that the circulatory arrest time was longer in the Non-Octogenarian group than in the Octogenarian group is reasonable. The Non-Octogenarian group had a significantly higher body mass index than the Octogenarian group (Table 1). Body mass index affects the thickness of subcutaneous fat and appears to affect the distance between the skin and distal anastomosis. In addition, the Non-Octogenarian group tended to have a higher rate of treating more dissecting aneurysms than the Octogenarian group, but this was not significant. In case of a dissecting aneurysm, trimming a distal anastomotic site takes more time than with a true aneurysm. Therefore, this situation may have also affected the circulatory arrest time.

Another major finding of the present study was that there was no significant difference in survival at a mean follow-up duration of 3.8 years between the 2 groups (Fig. 4). In addition, preoperative age ≥80 years was not an independent predictor of overall mortality in univariable Cox proportional hazard analysis (Table 4). This finding may be due to the fact that there were no differences in postoperative outcomes between the 2 groups. However, a difference appeared between the 2 survival curves 4 years after the operation (Fig. 4). A more significant difference in survival may appear as the follow-up time increases between the 2 groups. A larger validation study is required to clarify this relationship.

**Table 4:** Univariable cox hazard proportional model for the predictors of all-cause death

Predictor	HR	95% CI	*P*-value
Sex (male)	0.80	0.23–2.84	0.731
Body mass index (kg/m^2^)	0.95	0.78–1.15	0.608
Hypertension	0.54	0.18–1.58	0.261
Diabetes mellitus	0.54	0.12–2.42	0.423
Dyslipidaemia	0.63	0.20–1.98	0.427
Smoking history	0.72	0.26–2.02	0.530
COPD	2.36	0.53–10.55	0.260
Previous CVD	1.14	0.32–4.02	0.844
Peripheral artery disease	1.06	0.24–4.69	0.943
eGFR (ml/min/1.73 m^2^)	0.99	0.96–1.02	0.451
eGFR <60 ml/min/1.73 m^2^	1.34	0.49–3.7.	0.572
Previous sternotomy	0.91	0.21–4.04	0.901
Previous thoracic aortic surgery	0.75	0.10–5.47	0.748
Aneurysm diameter	0.69	0.94–1.09	0.686
Atherosclerotic aneurysm	1.31	0.45–3.84	0.621
Penetrating aortic ulcer	0.80	0.18–3.54	0.765
Chronic dissection	0.81	0.23–2.89	0.748
Operation time >300 min	1.16	0.33–4.15	0.814
CPB time >180 min	1.37	0.31–6.11	0.682
SCP time >100 min	0.35	0.08–1.54	0.164
Circulatory arrest time >60 min	1.02	0.29–3.61	0.980
Minimum temperature (°C)	1.06	0.84–1.33	0.615
Age ≥80 (years)	2.39	0.80–7.15	0.119
Age (years)	1.08	1.01–1.16	0.037

CI: confidence interval; COPD: chronic obstructive pulmonary disease; CPB: cardiopulmonary bypass; CVD: cerebrovascular disease; eGFR: estimated glomerular filtration rate; HR: hazard ratio; SCP: selective cerebral perfusion.

Old age still prevents many patients from being referred for cardiac surgery [17–20]. Indeed, TAR remains a high-risk operation requiring hypothermic circulatory arrest and prolonged use of CPB compared with other cardiac surgeries. However, in the present study, an age ≥80 years was not associated with early and mid-term outcomes postoperatively in patients who underwent TAR in our institution. Previous studies reported that an octogenarian was an independent predictor of hospital mortality by multivariable logistic regression analysis in patients undergoing TAR [13, 14]. These previous studies included patients who presented with acute type A dissection, aneurysmal rupture and shock. Therefore, the patients’ preoperative condition and complexity of the disease may have affected the postoperative outcomes. We were able to investigate the effect of age on postoperative outcomes by limiting the patients undergoing TAR to those who underwent isolated elective surgery. Our study suggests that open heart surgery should not be denied simply because of an age ≥80 years when performing isolated elective TAR.

The major concern associated with lower body circulatory arrest is paraplegia. In our institution, circulatory arrest was achieved when the tympanic temperature fell to 25°C and the core temperature fell to <30°C. The mean circulatory arrest time was 50.3 ± 21.6 min and the minimum core temperature was 25.1 ± 2.2°C in our cohort. Wang *et al.* investigated the perioperative outcomes in 1708 patients undergoing aortic arch surgery with hypothermic circulatory arrest and unilateral SCP [11]. These authors found that age was an independent risk factor of postoperative paraplegia and that the risk of paraplegia increased when the circulatory arrest time was longer than 40 min or nasopharyngeal temperature was greater than ∼24°C. However, no patients underwent postoperative paraplegia in the present study, which may have resulted in a good mid-term outcome in octogenarians. There are several plausible explanations for these results. In Wang *et al.*’s [11] study, TAR with a frozen elephant trunk was performed in 1419 (83.1%) patients. Frozen elephant trunk procedure was reported to increase the risk of postoperative paraplegia associated with occlusion and thromboembolism of the intercostal artery [21–23], which might have affected the postoperative outcomes. Additionally, we routinely performed three-vessel SCP with the aim of collateral perfusion. We previously reported that bilateral SCP partially perfused the thoracic cord via the collateral circulation from the vertebral arteries through an anterior spinal artery [24]. However, whether unilateral SCP can perfuse the thoracic cord as much as bilateral SCP, even in patients whose collateral circulation is poor, remains controversial. Our surgical treatment strategy may also have affected the incidence of paraplegia.

In the present study, 2 patients experienced stroke postoperatively (Table 2). One patient in the Octogenarian group had many plaques in the brachiocephalic artery preoperatively, and 1 in the Non-Octogenarian group had many plaques from the right common carotid artery to the right internal carotid artery. Previous studies reported that total arch repair through a median sternotomy caused postoperative stroke with rate of 2.5–5.8% [1, 3, 10]. In our entire cohort, postoperative stroke rate was 1.4% (2/140), which was better result compared to previous studies. During SCP procedure, we divided 3 brachiocephalic branches at the level of healthy arterial wall and inserted SCP cannula into the 3 vessels as carefully as possible. These methods may have associated with postoperative lower stroke rate.

It is important to assess preoperative frailty, cognition function and physical performance status especially in elderly patients undergoing open heart surgery. In our institution, we perform TAR for aortic arch aneurysm regardless of age without performing debranching thoracic endovascular aortic repair and do not routinely evaluate them. However, elderly patients often have multiple comorbidities such as muscle weakness and cognitive impairment which are not seen in young patients. To make the risk assessment of preoperative risk more accurate in octogenarian, it seems necessary to perform those tests.

### Limitations 

This was a retrospective study in a single centre. First, all enrolled subjects were Japanese patients, which limits the generalizability of the findings. Second, the total number of Octogenarians group was small (*n* = 30), and the number of patients followed for >4 years was small. There is an increased risk of a type II error. Finally, logistic regression analysis for postoperative stroke could not be performed because of the small number of events (2/140 patients).

## CONCLUSIONS

Preoperative age ≥80 years is not associated with worse postoperative outcomes after elective isolated TAR using mild hypothermic lower body circulatory arrest with bilateral antegrade SCP. Heart surgery should not be denied simply because of older age when performing isolated elective TAR.

**Conflict of interest:** none declared.

### Author contributions

**Kohei Hachiro:** Formal analysis; Investigation; Writing—original draft. **Takeshi Kinoshita:** Writing—review & editing. **Tomoaki Suzuki:** Writing—review & editing. **Tohru Asai:** Supervision.

### Reviewer information

Interactive CardioVascular and Thoracic Surgery thanks the anonymous reviewer(s) for their contribution to the peer review process of this article.

## ABBREVIATIONS

CI: Confidence interval
CPB: Cardiopulmonary bypass
HR: Hazard ratio
SCP: Selective cerebral perfusion
TAR: Total arch replacement
